# Enrichment with anti-cytokeratin alone or combined with anti-EpCAM antibodies significantly increases the sensitivity for circulating tumor cell detection in metastatic breast cancer patients

**DOI:** 10.1186/bcr2131

**Published:** 2008-08-07

**Authors:** Glenn Deng, Michael Herrler, David Burgess, Edward Manna, David Krag, Julian F Burke

**Affiliations:** 1 Biology, Genetix USA Inc, 120 Baytech Drive, San Jose, CA 95134, USA; 2 Vermont Cancer Center, University of Vermont, 89 Beaumont Avenue, Burlington, VT 05405, USA; 3 Biology, Genetix Ltd, Queensway, New Milton, Hampshire, BH25 5NN, UK

## Abstract

**Introduction:**

Circulating tumor cells (CTCs) are detectable in most cancer patients and they can meet an existing medical need to monitor cancer patients during a course of treatment and to help determine recurrent disease. CTCs are rarely found in the blood of cancer patients and enrichment is necessary for sensitive CTC detection. Most CTC enrichment technologies are anti-EpCAM antibody based even though CTC identification criteria are cytokeratin positive (CK^+^), CD45 negative (CD45^-^) and 4'6-diamidino-2-phenylindole (nuclear stain) positive (DAPI^+^). However, some tumor cells express low or no EpCAM. Here we present a highly sensitive and reproducible enrichment method that is based on binding to anti-CK alone or a combination of anti-CK and anti-EpCAM antibodies.

**Methods:**

Blood samples from 49 patients with metastatic breast cancer were processed using the CellSearch™ system (Veridex, LLC, Raritan, NJ, USA), in parallel with our CTC assay method. We used anti-CK alone or in combination with anti-EpCAM antibodies for CTC enrichment. Brightfield and fluorescence labeled anti-CK, anti-CD45 and DAPI (nuclear stain) images were used for CTC identification. The Ariol^® ^system (Genetix USA Inc, San Jose, CA, USA) was used for automated cell image capture and analysis of CTCs on glass slides.

**Results:**

Our method has the capability to enrich three types of CTCs including CK^+^&EpCAM^+^, CK^+^&EpCAM^-/low^, and CK^-/low^&EpCAM^+ ^cells. In the blind method comparison, our anti-CK antibody enrichment method showed a significantly higher CTC positive rate (49% vs. 29%) and a larger dynamic CTC detected range (1 to 571 vs. 1 to 270) than that of the CellSearch™ system in the total of 49 breast cancer patients. Our method detected 15 to 111% more CTCs than the CellSearch™ method in patients with higher CTC counts (>20 CTCs per 7.5 ml of blood). The three fluorescent and brightfield images from the Ariol^® ^system reduced the number of false-positive CTC events according to the established CTC criteria.

**Conclusion:**

Our data indicate that the tumor-specific intracellular CK marker could be used for efficient CTC enrichment. Enrichment with anti-CK alone or combined with anti-EpCAM antibodies significantly enhances assay sensitivity. The three fluorescent and brightfield superior images with the Ariol^® ^system reduced false-positive CTC events.

## Introduction

Circulating tumor cells (CTCs) are detectable in most blood samples from patients with metastatic cancer using different technologies. CTCs are rare and need to be enriched from the patients' blood sample for better detection [1,2]. CTC analysis has been performed mostly in breast cancer, the second leading cause of cancer death in women in the US and the Western world. Metastatic breast cancer occurs when tumor cells grow unregulated and eventually lose the ability to adhere to one another. Current models of metastasis support the idea that detached cancer cells travel in the lymphatic system, usually in axilla and intercostal spaces of the sentinel nodes, and/or in the blood system to a new site. Neo-vascularisation develops and a new tumor grows.

Bone is the most common site of metastasis in patients with breast cancer. Detecting tumor cells within bone marrow has emerged as a marker of disease recurrence or survival in breast cancer patients [3]. Braun *et al*. reported that 30% of women with primary breast cancer have disseminated tumor cells in their bone marrow. In a 10-year follow-up study, Braun *et al. *were able to show that these patients had a significantly decreased disease-free survival rate and overall survival rate when compared with patients with no disseminated tumor cells [4,5]. However, sampling of bone marrow is painful for the patient and aspiration cannot be used routinely for breast cancer monitoring.

Detection of CTCs in blood has obvious advantages as a non-invasive sampling procedure and has better potential of being a real-time biopsy of tumors because blood can be sampled frequently. Recently, Meng *et al*. analyzed CTCs from the blood of patients with newly diagnosed, advanced breast cancer and from patients with recurrent breast cancer by measuring gene status in CTCs compared with cancer cells in the primary tumor tissue [6]. It was concluded that individual tumor cell analysis could provide important information for clinical trials to test the correlations between gene status data obtained from CTCs before treatment and the responses of patients to various therapeutic regimens. This might lead to diagnostic tests that could select the therapy most likely to be effective for an individual patient. This could be an opportunity to evaluate CTCs as potential non-invasive tools for improving selection of individualised therapy [7].

Today, numerous methods are available to analyze CTCs from blood. Slide-based systems are the most commonly used. Traditionally, immunocytochemistry is combined with brightfield microscopy to detect CTCs on microscope slides. In 1999, a consortium of European laboratories participated in the first multi-centre study with the objective of reaching a general consensus on the criteria for defining a circulating epithelial cell as a cancer cell [8]. Subsequently, many new methods were developed that included improved immunomagnetic separation techniques. In addition, fluorescence-based assays gained importance. A review by Fehm *et al. *gives a good summary of the currently available CTC enrichment methods using slide-based detection assays [2].

CTCs are rarely found in the blood of cancer patients. Therefore, relatively large volumes of blood have to be processed to increase the sensitivity of the assay. Detection of CTCs without target cell enrichment or depletion of unwanted cells is challenging. Hsieh *et al. *described a high-speed scanning device that allows detection of circulating tumor cells after depletion of red blood cells [9]. However, a comparison to a reference method has yet to prove its superior performance. The well-studied CTC assays available today are based on enrichment with anti-EpCAM antibodies and subsequent detection with anti-cytokeratin (CK) [10,11]. The CellSearch™ system is one example of these assays; it is based on anti-EpCAM enrichment and is currently the only instrument with regulatory-approval that allows enumeration and characterization of CTCs in blood.

In a study by Cristofanilli *et al*., 177 patients with metastatic breast cancer were tested for the presence of CTCs using the CellSearch™ system. The study concluded that detection of CTCs before initiation of first-line therapy in patients with metastatic breast cancer is highly predictive of progression-free survival and overall survival [12,13]. Riethdorf *et al. *validated the CellSearch™ system in a multi-centre study and concluded that the system allows the reliable detection of CTCs in blood and is suitable for the routine assessment of metastatic breast cancer patients in the clinical laboratory [14].

CK is a specific tumor cell marker and is one criterion for CTC identification. Usually, anti-EpCAM antibody is used to enrich CTCs and CK is used to identify the cells. The differential expression of CK and EpCAM on the same cell will be the key to ensure that no target cells are missed. Those tumor cells that express CK but with low or no EpCAM, or vice versa [15-19], may not be enriched by anti-EpCAM antibody. Here we describe an improved anti-CK-based method for CTC enrichment and detection from the peripheral blood of breast cancer patients. Our method uses the same antibodies to enrich and identify CTCs. To compensate for low or no expression of EpCAM and CK, we also developed an assay with a combination of anti-CK and anti-EpCAM antibodies that allows the enrichment of all types of CTCs including CK^+^&EpCAM^+^, CK^+^&EpCAM^-/low ^and CK^-/low^&EpCAM^+ ^tumor cells. We developed a staining method that visualizes three fluorescent labeled markers and brightfield cell morphology information for CTC identification and successfully applied it to the Ariol^®^, an automated image capture and analysis system that can combine the three fluorescent and brightfield images on the same cell simultaneously. The comparison results with the CellSearch™ system have proven that our method has higher sensitivity, reproducibility and better accuracy.

## Materials and methods

The breast cancer cell lines MCF-7 and SK-BR-3 (ATCC, Rockville, MD, USA) were used for cell spiking experiments. Blood samples from healthy donors were obtained from Advanced Bioscience Resources, Inc. (Alameda, CA, USA). The healthy donors were between 18 and 50 years of age, and had no current or medical history of malignancy of epithelial origin. They were required to understand and sign an informed consent form that conforms to federal and institutional guidelines. Blood samples from breast cancer patients were obtained from the University of Vermont. All the metastatic breast cancer patients were over 18 years of age. All enrolled patients gave their informed consent for study inclusion and were enrolled using institutional review board-approved protocols. Patients must have had radiological or histological evidence of metastatic cancer with haemoglobin levels more than 10 gm% and haematocrit levels more than 30%. No patients were excluded from this study because of prior medical treatment.

Blood from each healthy donor was collected into EDTA tubes (BD Biosciences, San Jose, CA, USA) or CellSave tubes (Veridex, LLC, Raritan, NJ, USA). Blood samples were maintained at room temperature for different time intervals and processed within a maximum of 72 hours after blood drawing. For the methods comparison study, patient blood samples were shipped from the University of Vermont to Genetix USA, Inc. (San Jose, CA, USA) and Quest Diagnostics Nichols Institute respectively (Chantilly, VA, USA). Blood samples were processed in parallel in the blind study. A 7.5 to 10 ml sample of whole blood was transferred to a 50 ml Falcon tube (Corning, Lowell, MA, USA) and gently mixed with red blood cell removal buffer (CTC Enrichment and Detection Kit, Genetix, New Milton, UK). After approximately five to 10 minutes (when the colour of the blood changed to a transparent cherry red), cells were immediately centrifuged at 700 × g for 10 minutes at room temperature using a Beckman GS-6 centrifuge with GH-3.8 buckets. The cell pellet was carefully resuspended in 0.5 ml dilution buffer (Carcinoma Cell Enrichment and Detection Kit, Miltenyi Biotec, Bergisch Gladbach, Germany). Blocking, permeation and fixation reagents (CTC Enrichment and Detection Kit, Genetix, New Milton, UK) and enrichment reagents (Carcinoma Cell Enrichment and Detection Kit, Miltenyi Biotec, Bergisch Gladbach, Germany) were added as described in Genetix's instructions for the CTC Enrichment and Detection kit.

Cells were incubated for 45 minutes at room temperature and gently mixed every 10 minutes. Dilution buffer was added to each tube to make up a total of 10 ml in each. The tubes were centrifuged at 300 × g for 10 minutes at room temperature and the cells were resuspended in 1 ml dilution buffer. Each sample was carefully applied to the centre of one 0.5 ml dilution buffer equilibrated MS separation column (Miltenyi Biotec, Bergisch Gladbach, Germany) attached to a MiniMACS or OctoMACS separator (Miltenyi Biotec, Bergisch Gladbach, Germany). After washing three times with 0.5 ml dilution buffer, the columns were detached from the cell separator.

Target cells were eluted into a 5 ml tube with 1 ml dilution buffer. Hettich cytospin chambers (Hettich, Germany) were assembled and the eluted target cells were directly added into the cytospin reservoirs using a funnel that creates cytospins with a diameter of 8.7 mm. Target cells were deposited onto Poly-Prep™ PLL (poly-L-lysine coated) glass slides (Sigma, St. Louis, MO, USA) by centrifugation for three minutes at 800 rpm using a Hettich Universal 16 centrifuge (Hettich, Germany). The supernatant was carefully removed using a fine-tipped transfer pipette. The slides were centrifuged one more time for one minute at 1000 rpm to remove any extra liquid. The slides were taken out from cytospin and dried for 30 to 60 minutes at room temperature or 37°C using a slide warmer (Fisher, Pittsburgh, PA, USA). Target cells were fixed in 100% acetone (Sigma) at -20°C for 10 minutes. The slides were dried at room temperature for 30 minutes.

A Shandon Cadenza Immuno Stainer (Thermo Scientific, Waltham, MA, USA) was used for the target cell staining. The glass slides with fixed target cells were assembled onto the Immuno Stainer and washed with PBS buffer (Genetix, New Milton, UK) twice for three minutes. The slides were incubated for 30 minutes with Image iT FX signal enhancer (Invitrogen, Carlsbad, CA, USA). Cells were stained by either direct or indirect labeling antibodies. In the case of direct labeling, we used a set of fluorescein isothiocyanate (FITC) conjugated anti-mouse IgG1 antibodies recognising CK 8, 18 and 19 (CTC Enrichment and Detection Kit, Genetix, New Milton, UK); haematopoietic cells were stained with a DyeLight 549 conjugated anti-mouse IgG1 antibody recognising CD45 (CTC Enrichment and Detection Kit, Genetix, New Milton, UK). The specificity of the antibodies was tested by Western blot analysis and/or immunohistochemistry staining with specific tumor tissues.

The healthy donor's blood samples with no tumor cells were used as negative controls. The blood samples containing tumor cell were used as positive controls. The non-specific binding blocking reagents were used in the CTC assays.

In the case of indirect labeling, the primary antibody cocktail contained a mouse IgG2 anti-CK (CAM5.2, BD Biosciences, San Jose, CA, USA) and a mouse IgG1 anti-CD45 antibody (Lab Vision, Freemont, CA, USA). Slides were incubated for 30 minutes with directly labeled antibodies (anti-CK FITC, anti-CD45 TexasRed) or incubated for 30 minutes with primary antibodies (anti-CK and anti-CD45). They were subsequently washed twice with PBS buffer, then incubated with the secondary antibodies (goat anti-mouse Alexa 488 IgG2a for CK, goat anti-mouse Alexa 568 IgG1 for CD45; Invitrogen/Molecular Probes, San Diego, CA, USA) for another 30 minutes. After washing twice with PBS buffer, the slides were stained with brightfield staining dye (CTC Enrichment and Detection Kit, Genetix, New Milton, UK) to enable visualization of intact cell morphology under white light exposure. The 4'6-diamidino-2-phenylindole (DAPI) mounting medium (Vector Laboratories, Burlingame, CA, USA) was used for cell nuclei staining.

Image analysis was performed using the Ariol^® ^system. Slides of stained target cells had barcodes affixed to them and were introduced into the system via the data entry application. The Ariol^® ^was configured for all scanning, image capture and processing to be associated with the CTC assay. The cell presentation area on the slide was fully scanned with FITC, TexasRed and DAPI fluorescence channels and in brightfield. The images of CK-FITC-positive targets were captured and presented in the image gallery. Only the cells with CK and DAPI positive, and CD45 negative [10-15,20] were counted as CTCs. Cells were further identified by brightfield images that have smooth staining and a round shape to discriminate from debris or cell fragments.

All analyzed data was reviewed and re-classified as appropriate. A report included the selected cell images for the combined fluorescence channels (composite view), an image for each of the separate fluorescence channels and a brightfield image.

The effect of blood storage temperature and addition of a cell preservative on tumor cell stability was tested with tumor cell (GFP or DsRed MCF-7, DAPI labeled MCF-7 or unlabelled MCF-7 and SKBR-3) spiked blood samples. Samples were either drawn into regular EDTA tubes (Becton Dickinson, USA) or CellSave tubes that contain EDTA plus a cell preservative. Samples were tested immediately after blood draw or stored either for 24, 48 or 72 hours at room temperature or 4°C until processing. In total, 60 samples were processed, including 12 controls.

Our method was compared with the CellSearch™ system in a blinded experiment (CTCs were analyzed separately according to the criteria before knowing any results from CellSearch™) using blood samples from metastatic breast cancer patients. The patients' blood samples were obtained from the University of Vermont, and were drawn into two separate CellSave™ blood collection tubes. One tube of the blood was processed in our laboratory (Genetix, USA) in San Jose, CA; another tube of blood was processed by Quest Diagnostics Nichols Institute according to the CellSearch™ protocol the day after blood drawing. In total, 49 patient samples were tested using both methods. Fifty control samples from healthy donors were tested as negative controls.

## Results

### Blood collection, enrichment assay sensitivity and reproducibility

EDTA and CellSave tubes were used for blood collection and the spiked tumor cell recoveries were compared (Table 1). EDTA and CellSave tubes both showed very reliable cell recovery results at 4°C up to three days after blood collection, with the EDTA tubes being slightly better than CellSave, but with no significant difference. At room temperature, the CellSave tube was clearly better than the EDTA tube. The cell recovery from the CellSave tube was better than from the EDTA tube after the blood sample was stored at room temperature for more than one day. Table 2 shows the sensitivity and reproducibility of our CTC assay method. On day 1, the mean cell recovery rate was 74.9% (standard deviation [SD] = 6.0%, coefficient of variation [CV] = 8.1%); on day 2 the mean cell recovery rate was 80.6% (SD = 8.2%, CV = 10.1%); and finally on day 3, the mean cell recovery rate was 79.4% (SD = 9.8%, CV = 12.4%). There was no significant difference in the day to day assay repeats. The mean cell recovery rate for 16 replicates processed on the three different days was 78.9% (SD = 8.2%, CV = 10.4%) (Table 2).

**Table 1:** Spiked MCF-7 cell recovery rates in EDTA and CellSave tubes

	**At 4°C**				**At room temperature**			
	
	**0 to 4 hrs**	**24 hrs**	**48 hrs**	**72 hrs**	**0 to 4 hrs**	**24 hrs**	**48 hrs**	**72 hrs**
**EDTA tube**	63 ± 2	75 ± 1	73 ± 5	69 ± 7	51 ± 17	37 ± 1	29 ± 12	9 ± 5
**CellSave tube**	75 ± 12	61 ± 22	57 ± 9	70 ± 10	71 ± 9	59 ± 15	49 ± 3	39 ± 13

Results shown as mean ± standard deviation.

**Table 2:** Spiked MCF-7 cell recovery rates after enrichment from blood

**Day repeat**	**Number of repeats**	**Recovery (%) mean ± SD**	**% CV**
**Day 1**	3	74.9 ± 6.0	8.1
**Day 2**	5	80.6 ± 8.2	10.1
**Day 3**	8	79.9 ± 9.8	12.4
**Total**	16	78.9 ± 8.2	10.4

SD, standard deviation; CV, coefficient of variation.

### CTC detection in metastatic breast cancer patients

A total of 49 blood samples from metastatic breast cancer patients were tested with two CTC assay methods and the numbers of CTC detected from individual patients are shown in Table 3 (only shows the CTC-positive individuals either by CellSearch™ or our CTC method). With our method, 49% (24 out of 49) of metastatic breast cancer patients had at least one CTC in 7.5 ml blood. The CellSearch™ assay detected CTCs in only 29% of patients (14 out of 49, three patients' assays failed by Quest were counted as negative).

**Table 3:** Method comparison for circulating tumor cell (CTC) detection from blood samples of breast cancer patients

**Patient ID**	22	76	77	37	39	60	16	45	53	56	52	47	17	48	54	59	74	35	46	14	19	18	78	68	64	20	21
**CTC# (CellSearch™)**	1	F*	F*	0	0	0	2	0	0	2	3	7	0	0	0	1	1	2	0	4	12	0	F*	21	25	143	270
**CTC # (Ariol^®^)**	0	0	0	1	1	1	1	2	2	2	2	2	3	3	3	3	3	3	4	8	9	11	26	27	29	164	571

F* Assay failed by Quest CellSearch™

Our method had a significantly higher CTC detection rate than the CellSearch™ system (p < 0.01) and a wider dynamic CTC range (1 to 571 vs. 1 to 270). The number of patient samples reaching Immunicon's (current Veridex LLC, USA) prognostically relevant cut-off level of 5 or more CTCs in 7.5 ml blood with our method was slightly higher than with CellSearch™ (16% vs. 12%) but this was not significant (p = 0.396). The percentage of samples with one to four CTCs in our method was significantly higher than the CellSearch™ method (33% vs. 16%, p < 0.01) (Table 4). In the patients with higher CTC counts (>20 CTCs per 7.5 ml blood), our method detected 15% to 111% more CTCs than the CellSearch™ method at the individual level. No CTCs were detected in the 50 normal healthy donors (negative controls) with our method (data not shown).

**Table 4:** Circulating tumor cell (CTC) distribution

**CTC #**	**CellSearch™**	**Ariol^®^**
**0**	35 (71%)	25 (51%*)
**1 to 4**	8 (16%)	16 (33%*)
**≥ 5**	6 (12%)	8 (16%)
**Positive %**	29	49*

* Significant difference (p < 0.01) with Z-test.

**Figure 1:**
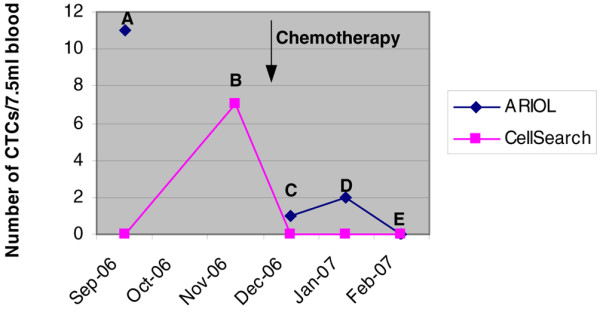
Tracking circulating tumor cell (CTC) counts from a metastatic breast cancer patient (ID# 18) in response to different therapies (Ariol^® ^vs. CellSearch™).

**Figure 2:**
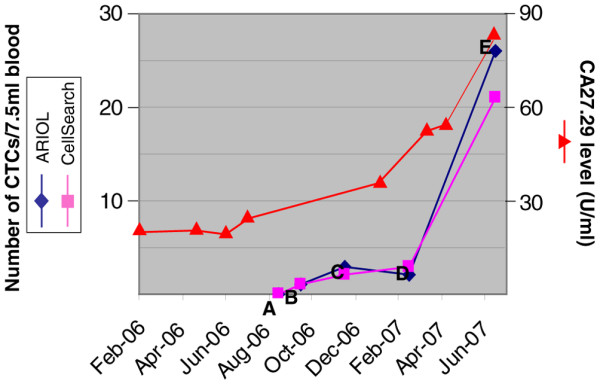
Tracking circulating tumor cell (CTC) counts from a metastatic breast cancer patient (ID# 68) in response to different therapies (Ariol^® ^vs. CellSearch™).

During the comparison study for our methods, we repeatedly collected blood samples from the same patients (Figures 1 and 2). Patient 18 (Figure 1) was a 45-year-old female with stage IV breast cancer, metastatic to bone and liver. At time point A and two weeks before time point B, the patient received two cycles of bevacizumab (Genentech, USA)/paclitaxel (Hospira, USA). Capecitabine (Roche, USA) chemotherapy was initiated at the beginning of November 2006 and in response the CTC count decreased to less than five CTCs and was 0 at February 2007. The CellSearch™ assay detected at time point B more than five CTCs. All other time points resulted in 0 CTCs. In contrast, our CTC assay, tracked CTCs from time point A over C, D, and E. We were not able to process the blood sample at time point B with our assay due to problems with the shipment of the sample.

Patient 68 (Figure 2) had stage IV breast cancer with extensive metastatases to bone. Initially, the patient was treated with tamoxifen (Generic), then weekly paclitaxel when the cancer progressed. The patient went through an incomplete course of radiation therapy. Letrozole (Novartis, USA) was initiated in response to an increasing level of CA27.29 with good response. However, CA27.29 levels increased again, which were reflected in the CTC counts (time point E). Both the CellSearch™ method and our Ariol^® ^CTC method were in concordance with the number of CTCs detected at time points A to E.

**Figure 3:**
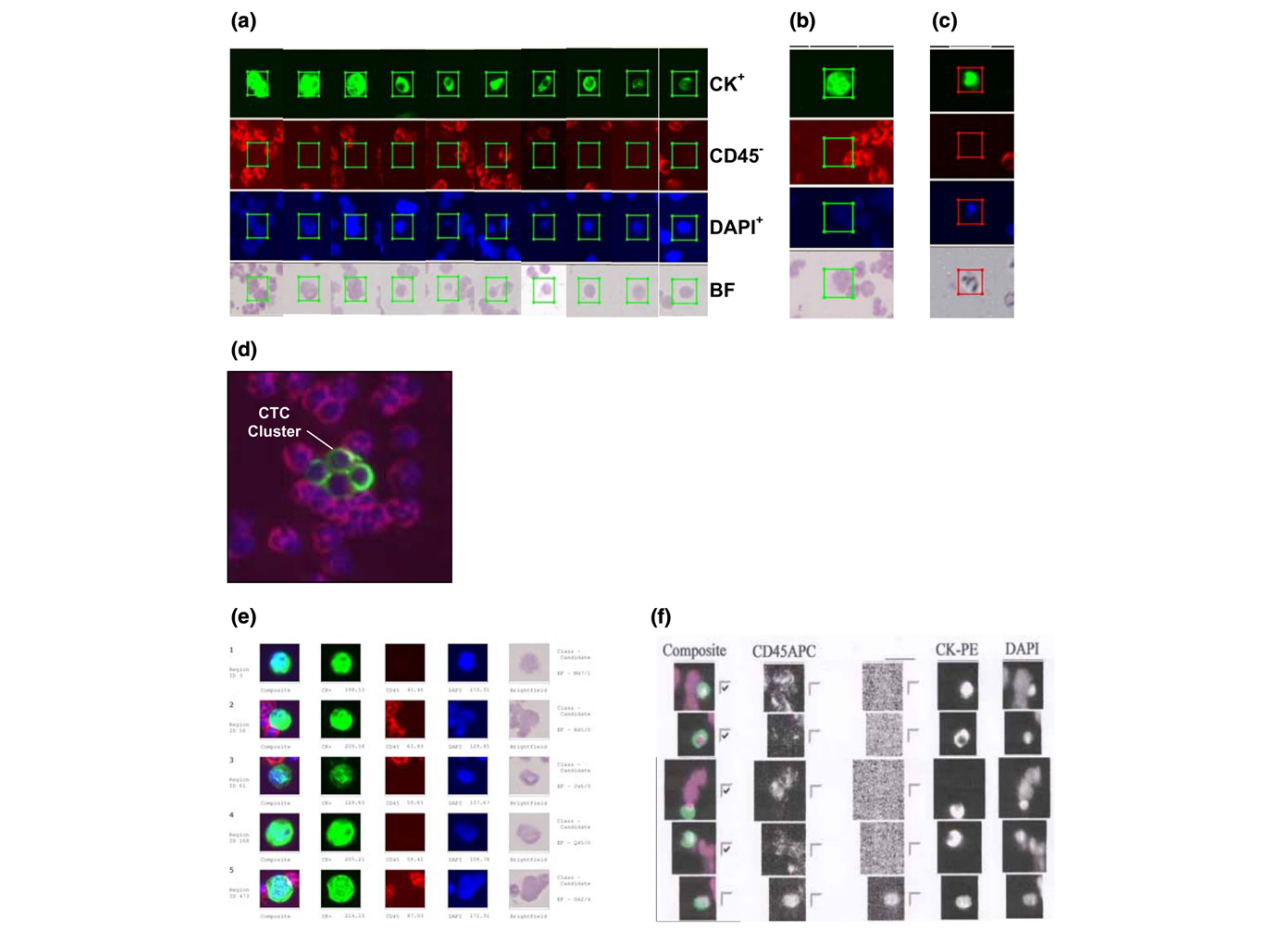
Image analysis of circulating tumor cells (CTCs) detected in the blood of breast cancer patients. **(a)** CTCs detected in blood samples from the same or different breast cancer patients vary in size, shape and cytokeratin (CK) expression level. **(b)** 'Boxed' cells with confirmed CTCs; to be classified as a CTC, the cell should be positive for CK, negative for CD45, positive for nuclear staining and be identified as intact cell through the brightfield image. **(c)** Brightfield (BF) imaging shows cell morphology, allowing discrimination between cells and artifacts like dye debris or cell fragments. **(d)** Composite image of a CTC cluster from a metastatic breast cancer patient. **(e) **Ariol^®^'s output report format including image information and such data as the signal level intensity, cell location in 'England Finder' (EF) coordinates and selected case information. **(f) **Image output report from the CellSearch™ system.

Figure 3 shows the imaging capability and quality of the Ariol^® ^system compared with the Cellsearch™ system. After deposition of enriched cells to microscope glass slides, cells were stained with fluorescent dye labeled antibodies and haematoxylin. The slides were automatically scanned and imaged in three fluorescent channels and brightfield with the Ariol^®^. We counted the cell as a CTC only when it was CK^+^, DAPI^+ ^and CD45^- ^[10-15,20] and with intact cellular morphology. Our method detected CTCs in blood samples from the same or different breast cancer patients with various sizes, shapes and CK expression levels (Figure 3(a)). In addition, the brightfield image shows the cell's morphology, allowing discrimination between intact cells and artifacts like debris or cell fragments (Figures 3(b) and 3(c)) and reduced false-positive CTC events in the assay. When the blood samples from the cancer patients were compared, our method and image analysis system could detect CTC clusters frequently and clearly show them in the gallery of the target image captures (Figure 3(d)), which the CellSearch™ system did not detect in this study. The output report from Ariol^® ^provides image information and data such as signal intensity, cell location in 'England Finder' (EF) co-ordinates and selected case information (Figure 3(e)). The image output report from the CellSearch^® ^system has three fluorescence channels capability but no brightfield information as shown in Figure 3(f).

### Combination of anti-CK and anti-EpCAM antibodies for CTC enrichment

**Figure 4:**
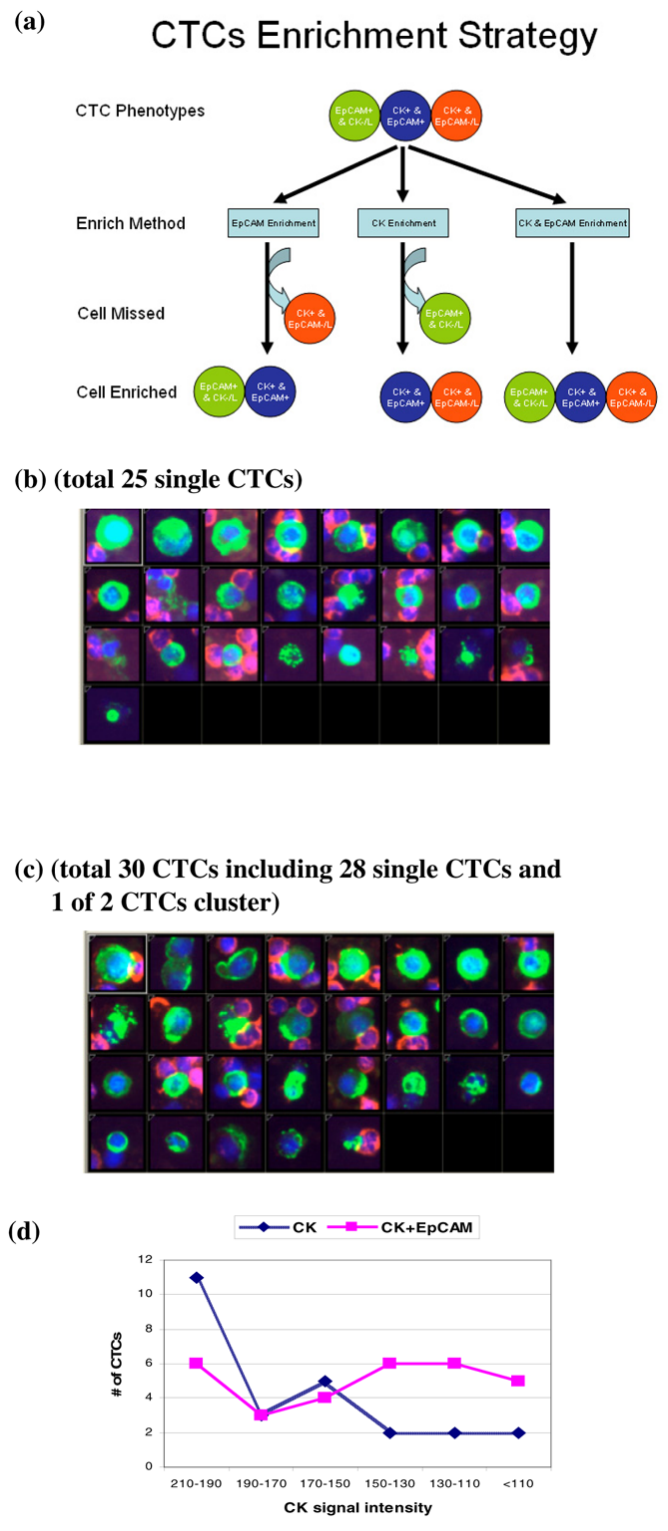
Circulating tumor cell (CTC) enrichment from blood of breast cancer patients using EpCAM and cytokeratin (CK) antibodies coupled to MACS microbeads. **(a)** CTCs enrichment strategy for three types of CTCs. This will be the mode to enrich all CTCs theoretically. **(b)** Image gallery showing CTCs recovered after enrichment with anti-CK antibody only; **(c)** Image gallery showing the higher number of CTCs recovered after enrichment with a combination of anti-CK and anti-EpCAM antibodies (the same blood sample as Figure 4(b)). **(d)** Plot showing CTC recovery rates after enrichment with anti-CK or a combination of anti-CK and anti-EpCAM in relationship to average cell anti-CK signal intensity.

The CTC-enrichment efficiency of anti-CK alone and the combination of anti-CK and anti-EpCAM antibodies was compared using the same patient's blood samples. Figure 4(a) shows our new strategy using a combination of microbeads coated with anti-CK and anti-EpCAM antibodies to enrich CTCs from patient blood samples. The CellSearch™ system represents an example of CTC enrichment with anti-EpCAM and detection with CK. Our current method uses anti-CK antibody-based enrichment to enrich and detect all CK^+ ^CTCs. Our new strategy is to use a combination of anti-CK and anti-EpCAM antibodies to enrich and detect all types of CTCs according to the current CTCs criteria.

We looked at the CTC detection using anti-CK (our current method) and anti-EpCAM enrichment (CellSearch™ method) in a blind comparison study. Here we also compared the CTC enrichment and detection using anti-CK alone and the combination of anti-CK and anti-EpCAM antibodies in blood samples from breast cancer patients. Figure 4 shows that in one breast cancer patient, 25 CTCs were detected (all single CTCs) in one tube of the blood using anti-CK microbeads and 30 CTCs were detected (28 single CTCs plus one two-CTC cluster) in another tube of the same patient's blood using the combination of anti-CK and anti-EpCAM antibodies microbeads (Figures 4(a), (b), (c)). A 20% higher CTC detection rate was achieved with our new strategy.

By comparing the average CK intensity of the enriched CTCs, the enrichment with the combination antibodies (anti-CK and anti-EpCAM) showed not only a higher CTC count, but also detected more CTCs with relatively low CK expression levels (Figure 4(d)). This result confirmed the theory that the method using a combination of antibodies (anti-CK and anti-EpCAM) had the highest sensitivity, followed by anti-CK enrichment and then anti-EpCAM enrichment.

## Discussion

The three criteria for CTCs determination are considered to be CK^+^, DAPI^+ ^and CD45^- ^[10-15,20]. CTCs are rare and heterogeneous in blood. Many different methods have been developed to isolate CTCs from peripheral blood and disseminated tumor cells from bone marrow [2,21,22]. Today, available methodologies include density gradient centrifugation [23-31], immunomagnetic cell enrichment [32-39] and/or depletion [40-42], flow cytometry [43,44], filtration [45-47] and more. Often, different methods are combined with each other, such as density gradient centrifugation and immunomagnetic cell enrichment and/or depletion.

The CellSearch™ is automated, uses standardized reagents and was recently validated in a multi-centre study [14]. The study was conducted at three independent laboratories to validate the analytical performance of the system for clinical use in patients with metastatic breast cancer. It was concluded that the system enabled the reliable detection of CTCs in blood to routinely assess metastatic breast cancer patients. One of the limitations of the CellSearch™ system is the anti-EpCAM antibody-based enrichment strategy. Several authors reported the heterogeneous expression of EpCAM in mammary carcinomas [15,19]; downregulation of EpCAM was reported for disseminated tumor cells in bone marrow and CTCs in peripheral blood [15,19,48].

Most other reported CTC enrichment methods are also based on anti-EpCAM antibodies only, but the enriched CTCs are identified by anti-CK antibodies [10-15,20]. The reason for using anti-EpCAM antibodies to enrich CTCs is because EpCAM is a cell-surface marker and has an advantage in the cell enrichment process. Since EpCAM is not equally expressed in all tumor cells [15,19], ideally, it would make better sense to use the same antibodies to enrich and identify CTCs. In the present study, we described an anti-CK antibody-based CTC enrichment method and successfully applied it to CTC assays of patients' blood samples.

In the blind comparison study of our method compared with the CellSearch™ method, we used the same CellSave blood collection tubes, processed the same patients' blood samples simultaneously in our laboratory with our method and in Quest Laboratories with the CellSearch™ method. A higher positive CTC detection rate and a larger CTC detectable dynamic range were obtained from our method in the 49 breast cancer patients. We found that in the patients with low CTC counts, there was a good correlation between the two methods. However, there were also variations in which some patients were CTC positive by our method but not by CellSearch™, or vice versa. These variations may be a result of the low frequency of CTCs or the heterogeneity of CK and EpCAM expression in the CTCs, resulting in certain types of CTCs being missed by one or the another enrichment methods. In the 49 patient samples with CTC counts in the range of one to four, our method showed a significantly higher CTC detection rate (p < 0.01) and a larger CTC detection range than that of the CellSearch™ method. These results suggest that anti-CK antibody-based enrichment is more sensitive than anti-EpCAM antibody-based enrichment.

The most interesting results were obtained from the patients with higher CTC counts. In the patients with more than 20 CTCs, our anti-CK enrichment method showed a 15% to 111% higher sensitivity of CTC detection than that of the CellSearch™ method consistently in all four available patients. In other words, the anti-EpCAM antibody-based enrichment method could lose a portion of the real CTCs population in individual patients during the CTC assay process. The potential CTC loss by anti-EpCAM antibody-based enrichment could be up to 52.7% when compared with anti-CK antibody-based enrichment and could reduce the accuracy for patient CTC monitoring. Similar results were reported in bone marrow samples involving a total of 26 CK^+ ^tumor cells, of which none of them co-expressed EpCAM after chemotherapy. Most soft tissue tumors and all lymphomas were EpCAM negative [15-19,48]. More research is required to determine if those tumor markers' expression changes are related to cell growth or apoptosis [49]. In the case of CTCs that express low or no EpCAM and low or no CK, we need different strategies to enrich and target the correct cells.

CK is an intracellular protein. The biological mechanism of how this protein can be used for cell enrichment is not entirely understood. Our hypothesis is that the three reactions of cell permeation, anti-CK antibody binding to CK antigens and cell fixation are balanced in the same optimized reaction time period. Anti-CK antibodies penetrate into the cell and bind to CK inside the cell while the microbeads that are coupled to the antibodies remain outside the cell. The cells are permeabilised so anti-CK antibodies are able to bind to CK but not break the cells that will be the key to success. Usually intracellular markers can be more specific; in addition, they can be used alone or in combination with cell-surface markers for better cell enrichment. Sometimes, cell-surface markers are not available or may not be strong enough to enrich cells.

The key to the success of the current study lies with CK enrichment with the intracellular marker that when utilized alone showed equal or better sensitivity than surface-marker EpCAM, which makes the enrichment strategy using combination antibodies meaningful. When the anti-CK antibodies are added to the EpCAM enrichment process, it will be a plus effect. Because CK and EpCAM are both heterogeneously expressed on tumor cells (Figures 3 and 4(d)) [15,19,48], the enrichment with the combined antibodies may increase the sensitivity and reproducibility by the compensation effect of biomarkers from each other. CTCs can be classified into three classes: EpCAM^+^&CK^-/low^, EpCAM^-/low^&CK^+ ^and EpCAM^+^&CK^+^. CK^-^&EpCAM^- ^tumor cells are not discussed in this enrichment and detection study. None of the enrichment methods reported have the capability to enrich all three types of CTCs, either because of the enrichment antibody selection or the identification antibody selection, which will cause incomplete CTC profiling (the composition of different CTC types) and CTC downstream analysis for individual patients [6,16,29,35,50].

Our new CTC enrichment strategy uses the combination of anti-CK and anti-EpCAM antibodies and can theoretically enrich all the three types of CTCs by choosing the correct markers for detection (Figure 4(a)). Our results indicate that a higher CTC detection rate can be obtained by enriching with anti-EpCAM and anti-CK in combination than with anti-CK antibody alone. They also showed that the anti-EpCAM antibody may compensate for low CK expression, giving a better sensitivity and reproducibility for CTC detection in the new strategy (Figure 4(d)). Since most of the CTCs express both CK and EpCAM [51], this new CTC-enrichment strategy will consistently enrich most CTCs with CK^+^&EpCAM^+^, CK^-/low^&EpCAM^+ ^or CK^+^&EpCAM^-/low^. Therefore, this strategy may allow enrichment of CTCs for further CTC profiling and downstream analysis and may also be beneficial for the isolation and detection of CTC clusters (Figure 4(c)), which is another advantage in the clinical application [23,24].

During the blind methods comparison study, we found some events on the glass slide samples that meet the three CTC criteria but it was clearly not a full cell by brightfield image analysis. Reports using the CellSearch™ system describe a five CTC threshold for prognostic relevance. It is not clear that the presence of five CTCs has a clinical significance from 0 or one to four CTCs, or if the threshold is due to the sensitivity and accuracy of the CellSearch™ system. It is clear that no CTCs were detected from the blood samples of most normal individuals [10] and some CTCs are not easy to determine if the rely only on the three fluorescence channels analysis. Our method with the Ariol^® ^system provides additional brightfield cell morphological information that can reduce false-positive CTC events (Figures 3(b), (c)); this may provide additional information to understand the reasoning for the five CTC threshold for prognostic relevance.

Most recently, Nagrath et al. described a microchip technology that detected CTCs from almost 100% of patients with various cancers [1]. It is a very exciting study result, but it does not seem to solve the issue of missing the EpCAM^-/low^&CK^+ ^CTCs because their microchip uses the anti-EpCAM antibody-based enrichment method. There is no data on a direct comparison between the microchip technology and the CellSearch™ system, so it is not clear how much difference in sensitivity it will make compared with the current Food and Drug Administration cleared CellSearch™ system. To our knowledge, our method with the Ariol^® ^system is the first one to be directly compared with the CellSearch™ system and yield superior functionality in CTC enrichment and detection. The strength of our system is a multiplex anti-CK/anti-EpCAM-enrichment protocol combined with a fully automated image analysis platform using both fluorescent and brightfield detection. This method has the capability to enrich all three types of CTCs for complete CTC profiling and downstream analysis. The advantages of our method are obvious: significantly higher detection of heterogeneous CTCs, better image quality with fluorescent and brightfield images, and better detection of CTC clusters. A fully automated system with these advantages may benefit CTC-related studies and further CTC downstream analysis (fluorescence in situ hybridization, DNA/RNA etc) [35,50,52-59].

## Conclusion

The anti-CK antibody-based CTC-enrichment method uses an intracellular CK protein marker to enrich CTCs and achieve better sensitivity of CTC detection than that of the surface EpCAM protein marker in blood samples from breast cancer patients. The higher CTC detection in patients with high CTC counts using our method compared with the CellSearch™ system indicates the possible advantages of monitoring, diagnostics and complete CTC profiling. With the combination of anti-CK and anti-EpCAM antibodies, our method has the capability to enrich and detect most types of CTCs, improving the sensitivity and reproducibility for CTC enrichment and detection and providing complete CTC profiling and downstream analysis. Our method with the Ariol^® ^system provides superior cell image quality by combining fluorescent and brightfield images that allow discrimination between intact cells and cell fragments to reduce false-positive counts. The strategy of our method can be used for enrichment of other cell types with specific intracellular protein markers.

## Abbreviations

CK = cytokeratin; CTC = circulating tumor cell; CV = coefficient of variation; DAPI = 4'6-diamidino-2-phenylindole; FITC = fluorescein isothiocyanate; SD = standard deviation.

## Competing interests

GD and JFB are employed by Genetix and JFB holds Genetix's shares. MH and DB are former employees of Genetix. EM and DK are collaborators with Genetix and Genetix paid for some breast cancer patient blood samples. Genetix paid the article processing charge.

## Authors' contributions

All authors contributed to the preparation of the manuscript. GD and MH designed the overall study; carried out the experiments; were responsible for software testing, data collection, analysis and summary; and wrote the manuscript. DB programmed the software for CTC application and data collection. EM and DK participated in protocol development and interpretation of data. DK participated in the clinical study design, coordinated clinical patient sample collection and IRB protocol approval. JFB made the final decision to submit the manuscript for publication. All authors read and approved the final manuscript.

## References

[B1] Nagrath S, Sequit LV, Maheswaran S, Bell DW, Irimia D, Ulkus L, Smith MR, Kwak EL, Digumarthy S, Muzikansky A, Ryan P, Balis UJ, Tompkins RG, Haber DA, Toner M (2007). Isolation of rare circulating tumor cells in cancer patients by microchip technology. Nature.

[B2] Fehm T, Solomayer EF, Meng S, Tucker T, Lane N, Wang J, Gebauer G (2005). Methods for isolating circulating epithelial cells and criteria for their classification as carcinoma cells. Cytotherapy.

[B3] Pantel K, Brakenhoff RH (2004). Dissecting the metastatic cascade. Nat Rev Cancer.

[B4] Braun S, Pantel K, Mueller P, Janni W, Hepp F, Kentenich CRM, Gastroph S, Wischnik A, Dimpfl T, Kindermann G, Riethmuller G, Schlimok G (2000). Cytokeratin-positive cells in the bone marrow and survival of patients with stage I, II, or III breast cancer. N Engl J Med.

[B5] Braun S, Vogl FD, Naume B, Janni W, Osborne MP, Coombes RC, Schlimok G, Diel IJ, Gerber B, Gebauer G, Pierga JY, Marth C, Oruzio D, Wiedswang G, Solomayer EF, Kundt G, Strobl B, Fehm T, Wong GYC, Bliss J, Vincent-Solomon A, Pantel K (2005). A pooled analysis of bone marrow micrometastasis in breast cancer. N Engl J Med.

[B6] Meng S, Tripathy D, Shete S, Ashfaq R, Saboorian H, Haley B, Frenkel E, Euhus D, Leitch M, Osborne C, Clifford E, Perkins S, Beitsch P, Khan A, Morrison L, Herlyn D, Terstappen LWMM, Lane N, Wang J, Uhr J (2006). uPAR and HER-2 gene status in individual breast cancer cells from blood and tissues. Proc Natl Acad Sci USA.

[B7] Christofanilli M, Mendelsohn J (2006). Circulating tumor cells in breast cancer: Advanced tools for "tailored" therapy?. Proc Natl Acad Sci USA.

[B8] Borgen E, Naume B, Nesland JM, Kvalheim G, Beiske K, Fodstad O, Diel I, Solomayer EF, Theocharous P, Coombes RC, Smith BM, Wunder E, Marolleau JP, Garcia J, Pantel K (1999). Standardization of the immunocytochemical detection of cancer cells in BM and blood. I. Establishment of objective criteria for the evaluation of immunostained cells. Cytotherapy.

[B9] Hsieh HB, Marrinucci D, Bethel K, Curry DN, Humphrey M, Krivacic RT, Kroener J, Kroener L, Ladanyi A, Lazarus N, Kuhn P, Bruce RH, Nieva J (2006). High speed detection of circulating tumor cells. Biosens Bioelectron.

[B10] Allard WJ, Matera J, Miller MC, Repollet M, Connelly MC, Rao C, Tibbe AGJ, Uhr JW, Terstappen LWMM (2004). Tumor cells circulate in the peripheral blood of all major carcinomas but not in healthy subjects or patients with nonmalignant diseases. Clin Cancer Res.

[B11] Tibbe AGJ, Miller MC, Terstappen LWMM (2007). Statistical considerations for enumeration of circulating tumor cells. Cytometry A.

[B12] Cristofanilli M, Budd GT, Ellis MJ, Stopeck A, Matera J, Miller MC, Reuben JM, Doyle GV, Allard WJ, Terstappen LWMM, Hayes DF (2004). Circulating tumor cells, disease progression, and survival in metastatic breast cancer. N Engl J Med.

[B13] Cristofanilli M, Hayes DF, Budd GT, Ellis MJ, Stopeck A, Reuben JM, Doyle GV, Matera J, Allard WJ, Miller MC, Fritsche HA, Hortobagyi GN, Terstappen LWMM (2005). Circulating tumor cells: a novel prognostic factor for newly diagnosed metastatic breast cancer. J Clin Oncol.

[B14] Riethdorf S, Fritsche H, Muller V, Rau T, Schindlebeck C, Rack B, Janni W, Coith C, Beck K, Janicke F, Jackson S, Gornet T, Cristofanilli M, Pantel K (2007). Detection of circulating tumor cells in peripheral blood of patients with metastatic breast cancer: A validation study of the CellSearch system. Clin Cancer Res.

[B15] Thurm H, Ebel S, Kentenich C, Hemsen A, Riethdorf S, Coith C, Wallwiener D, Braun S, Oberhoff C, Janicke F, Pantel K (2003). Rare expression of epithelial cell adhesion molecule on residual micrometastatic breast cancer cells after adjuvant chemotherapy. Clin Cancer Res.

[B16] Woelfle U, Cloos J, Sauter G, Riethdorf L, Janicke F, van Diest P, Brakenhoff R, Pantel K (2003). Molecular signature associated with bone marrow micrometastasis in human breast cancer. Cancer Res.

[B17] Woelfle U, Sauter G, Santjer S, Brakenhoff R, Pantel K (2004). Down-regulated expression of cytokeratin 18 promotes progression of human breast cancer. Clin Cancer Res.

[B18] Willipinski-Stapelfeldt B, Riethdorf S, Assmann V, Woelfle U, Rau T, Sauter G, Heukeshoven J, Pantel K (2005). Changes in cytoskeletal protein composition indicative of an epithelial-mesenchymal transition in human micrometastatic and primary breast carcinoma cells. Clin Cancer Res.

[B19] Went PTH, Lugli A, Meier S, Bundi M, Mirlacher M, Sauter G, Dirnhofer S (2004). Frequent EpCam protein expression in human carcinomas. Hum Pathol.

[B20] Krag DN, Kusminsky R, Manna E, Ambaye A, Weaver DL, Harlow SP, Covelli M, Stanley MA, McCahill L, Ittleman F, Leavitt B, Krag M (2005). The detection of isolated tumor cells in bone marrow comparing bright-field immunocytochemistry and multicolor immunofluorescence. Ann Surg Oncol.

[B21] Zeidman I (1961). The fate of circulating tumor cells. I. Passage of cells through capillaries. Cancer Res.

[B22] Fidler IJ (1970). Metastasis: Quantitative analysis of distribution and fate of tumor emboli labeled with 125I-5-iodo-2'-deoxyuridine. J Natl Cancer Inst.

[B23] Brandt B, Junker R, Griwatz C, Heidl S, Brinkmann O, Semjonow A, Assmann G, Zanker KS (1996). Isolation of prostate-derived single cells and cell clusters from human peripheral blood. Cancer Res.

[B24] Schmidt H, De Angelis G, Bettendorf O, Eltze E, Semjonow A, Knichwitz G, Brandt B (2004). Frequent detection and immunophenotyping of prostate-derived cell clusters in the peripheral blood of prostate cancer patients. Intl J of Biological Markers.

[B25] Muller V, Stahmann N, Riethdorf S, Rau T, Zabel T, Goetz A, Janicke F, Pantel K (2005). Circulating tumor cells in breast cancer: correlation to bone marrow micrometastases, heterogeneous response to systemic therapy and low proliferative activity. Clin Cancer Res.

[B26] Liberti PA, Rao CG, Terstappen LWM (2001). Optimization of ferrofluids and protocols for the enrichment of breast tumor cells in blood. J Magnetism Magnetic Materials.

[B27] Wiedswang G, Borgen E, Schirmer C, Karesen R, Kvalheim G, Nesland JM, Naume B (2006). Comparison of the clinical significance of occult tumor cells in blood and bone marrow in breast cancer. Intl J Cancer.

[B28] Balic M, Dandachi N, Hofmann G, Hofmann G, Samonigg H, Loibner H, Obwaller A, Kooi A van der, Tibbe AGJ, Doyle GV, Terstappen LWMM, Bauernhofer T (2005). Comparison of two methods for enumerating circulating tumor cells in carcinoma patients. Cytometry B (Clin Cytom).

[B29] Baker MK, Mikhitarian K, Osta W, Callahan K, Hoda R, Brescia F, Kneuper-Hall R, Mitas M, Cole DJ, Gillanders WE (2003). Molecular detection of breast cancer cells in the peripheral blood of advanced-stage breast cancer patients using multimarker real-time reverse transcription-polymerase chain reaction and a novel porous barrier density gradient centrifugation technology. Clin Cancer Res.

[B30] Gertler R, Rosenberg R, Fuehrer K, Dahm M, Nekarda H, Siewert JR (2003). Detection of circulating tumor cells in blood using an optimized density gradient centrifugation. Recent Results Cancer Res.

[B31] Rosenberg R, Gertler R, Friederichs J, Fuehrer K, Dahm M, Phelps R, Thorban S, Nekarda H, Siewert JR (2002). Comparison of two density gradient centrifugation systems for the enrichment of disseminated tumor cells in blood. Cytometry.

[B32] Racila E, Euhus D, Weiss AJ, Rao C, McConnell J, Terstappen LWMM, Uhr JW (1998). Detection and characterization of carcinoma cells in the blood. Proc Natl Acad Sci USA.

[B33] Witzig TE, Bossy B, Kimlinger rant C, Donohue J, Suman VJ, Harrington D, Torre-Bueno J, Bauer KD (2002). Detection of circulating cytokeratin-positive cells in the blood of breast cancer patients using immunomagnetic enrichment and digital microscopy. Clin Cancer Res.

[B34] Krag DN, Ashikaga T, Moss TJ, Kusminsky RE, Feldman S, Carp NZ, Moffat FL, Beisch PD, Frazier TG, Gaskin TA, Shook JW, Harlow SP, Weaver DL (1999). Breast cancer cells in the blood: a pilot study. Breast J.

[B35] Meng S, Tripathy D, Shete S, Ashfaq R, Haley B, Perkins S, Beitsch P, Khan A, Euhus D, Osborne C, Frenkel E, Hoover S, Leitch M, Clifford E, Vitetta E, Morrison L, Herlyn D, Terstappen LWMM, Fleming T, Fehm T, Tucker T, Lane N, Wang J, Uhr J (2004). HER-2 gene amplification can be acquired as breast cancer progresses. Proc Natl Acad Sci USA.

[B36] Griwatz C, Brandt B, Assmann G, Zanker KS (1995). An immunological enrichment method for epithelial cells from peripheral blood. J Immunol Methods.

[B37] Brooimans RA, de Leeuw N, Bontenbal M, Gratama JW (2005). An immunomagnetic epithelial tumor cell enrichment model for minimal residual disease detection of cytokeratin 8+ malignancies. J Biol Regul Homeost Agents.

[B38] Choesmel V, Anract P, Hoifodt H, Thiery JP, Blin N (2004). A relevant immunomagnetic assay to detect and characterize epithelial cell adhesion molecule-positive cells in bone marrow from patients with breast carcinoma: immunomagnetic purification of micrometastases. Cancer.

[B39] Hu XC, Wang Y, Shi DR, Loo TY, Chow LWC (2003). Immunomagnetic tumor cell enrichment is promising in detecting circulating breast cancer cells. Oncology.

[B40] Wang ZP, Eisenberger MA, Carducci MA, Partin AW, Scher HI, Ts'o POP (2000). Identification and characterization of circulating prostate carcinoma cells. Cancer.

[B41] Meye A, Bilkenroth U, Schmidt U, Fussel S, Robel K, Melchior AM, Blumke K, Pinkert D, Bartel F, Linne C, Taubert H, Wirth MP (2002). Isolation and enrichment of urologic tumor cells in blood samples by a semi-automated CD45 depletion autoMACS protocol. Int J Oncol.

[B42] Taubert H, Blumke K, Bilkenroth U, Meye A, Kutz A, Bartel F, Lautenschlager C, Ulbrich EJ, Nass N, Holzhausen HJ, Koelbl H, Lebrecht A (2004). Detection of disseminated tumor cells in peripheral blood of patients with breast cancer: correlation to nodal status and occurrence of metastases. Gynecol Onco.

[B43] Allan AL, Vantyghem SA, Tuck AB, Chambers AF, Chin-Yee IH, Keeney M (2005). Detection and quantification of circulating tumor cells in mouse models of human breast cancer using immunomagnetic enrichment and multiparameter flow cytometry. Cytometry A.

[B44] Cruz I, Ciudad J, Cruz JJ, Ramos M, Gomez-Alonso A, Adansa JC, Rodriguez C, Orfao A (2005). Evaluation of multiparameter flow cytometry for the detection of breast cancer tumor cells in blood samples. Am J Clin Pathol.

[B45] Kahn HJ, Presta A, Yang LY, Blondal J, Trudeau M, Lickley L, Holloway C, McCready DR, Maclean D, Marks A (2004). Enumeration of circulating tumor cells in the blood of breast cancer patients after filtration enrichment: correlation with disease stage. Breast Cancer Res Treat.

[B46] Vona G, Sabile A, Louha M, Sitruk V, Romana S, Schutze K, Capron F, Franco D, Pazzagli M, Vekemans M, Lacour B, Brechot C, Paterlini-Brechot P (2000). Isolation by size of epithelial tumor cells: a new method for the immunomorphological and molecular characterization of circulating tumor cells. Am J Pathol.

[B47] Wong NS, Kahn HJ, Zhang L, Oldfield S, Yang LY, Marks A, Trudeau ME (2006). Prognostic significance of circulating tumor cells enumerated after filtration enrichment in early and metastatic breast cancer patients. Breast Cancer Res Treat.

[B48] Rao CG, Chianese D, Doyle GV, Miller MC, Russell T, Sanders RA, Terstappen LW (2005). Expression of epithelial cell adhesion molecule in carcinoma cells present in blood and primary and metastatic tumors. Intl J Oncol.

[B49] Mehes G, Witt A, Kubista E, Ambros PF (2001). Circulating breast cancer cells are frequently apoptotic. Am J Pathol.

[B50] Schmidt H, DeAngelis G, Eltze E, Gockel I, Semjonow A, Brandt B (2006). Asynchronous growth of prostate cancer is reflected by circulating tumor cells delivered from distinct, even small foci, harboring loss of heterozygosity of the PTEN gene. Cancer Res.

[B51] Pachmann K, Camara O, Kavallaris A, Schneider U, Schunemann S, Hoffen K (2005). Quantification of the response of circulating epithelial cells to neodadjuvant treatment for breast cancer: a new tool for therapy monitoring. Breast Cancer Res.

[B52] Smirnov DA, Zweitzig DR, Foulk BW, Miller MC, Doyle GV, Pienta KJ, Meropol NJ, Meiner LM, Cohen SJ, Moreno JG, Connelly MC, Terstappen LWMM, O'Hara SM (2005). Global gene expression profiling of circulating tumor cells. Cancer Res.

[B53] Smirnov DA, Foulk BW, Doyle GV, Connelly MC, Terstappen LWMM, O'Hara SM (2006). Global expression profiling of circulating endothelial cells in patients with metastatic carcinomas. Cancer Res.

[B54] Ge M, Shi D, Wu Q, Wang M, Li L (2005). Fluctuation of circulating tumor cells in patients with lung cancer by real-time fluorescent quantitative-PCR approach before and after radiotherapy. J Cancer Res Ther.

[B55] Lin JC, Chen KY, Wang WY, Jan JS, Liang WM, Wei YH (2002). Evaluation of cytokeratin-19 mRNA as a tumor marker in the peripheral blood of nasopharyngeal carcinoma patients receiving concurrent chemoradiotherapy. Int J Cancer.

[B56] Pelkey TJ, Frierson HF, Bruns DE (1996). Molecular and immunological detection of circulating tumor cells and micrometastases from solid tumors. Clin Chem.

[B57] Sabbatini R, Federico M, Morselli M, Depenni R, Cagossi K, Luppi M, Torelli G, Silingardi V (2000). Detection of circulating tumor cells by reverse transcriptase polymerase chain reaction of maspin in patients with breast cancer undergoing conventional-dose chemotherapy. J Clin Oncol.

[B58] Uchikura K, Takao S, Nakajo A, Miyazono F, Nakashima S, Tokuda K, Matsumoto M, Shinchi H, Natsugoe S, Aikou T (2002). Intraoperative molecular detection of circulating tumor cells by reverse transcription-polymerase chain reaction in patients with biliary-pancreatic cancer is associated with hematogenous metastasis. Ann Surg Oncol.

[B59] O'Hara SM, Moreno JG, Zeitzig DR, Gross S, Gomella LG, Terstappen LWMM (2004). Multigene reverse transcription-PCR profiling of circulating tumor cells in hormone-refractory prostate cancer. Clin Chem.

